# Exploring research advances and future trends in drug resistance in multiple myeloma: A comprehensive bibliometric analysis

**DOI:** 10.1097/MD.0000000000044279

**Published:** 2025-09-05

**Authors:** Xuanyu Yang, Fangzhen Lin, Siteng Zheng, Ye Gao, Zhengyang Li, Yuchen Mei, Yifan Xie, Jiayu Ke, Ling Ling

**Affiliations:** a School of Ophthalmology and Optometry, Jiangxi Medical College, Nanchang University, Nanchang, Jiangxi, People’s Republic of China; b The Second Clinical Medical College, Jiangxi Medical College, Nanchang University, Nanchang, Jiangxi, China; c The First Affiliated Hospital of Nanchang University, Nanchang, Jiangxi, China; d School of Food Science and Technology, Nanchang University, Nanchang, Jiangxi, China; e Nanchang Bright Eye Hospital, Nanchang, Jiangxi, China.

**Keywords:** bibliometric analysis, cancers, drug resistance, multiple myeloma, visualization

## Abstract

**Introduction:**

This bibliometric analysis aims to explore global trends, research hotspots, and future directions in multidrug resistance of multiple myeloma (MM), providing insights for overcoming resistance mechanisms and optimizing therapeutic strategies.

**Methods:**

We analyzed 3300 publications indexed in the Web of Science Core Collection (2015–2024) using CiteSpace and VOSviewer. Multidimensional evaluations of countries/regions, institutions, authors, journals, and keywords were conducted, supplemented by visual network mapping to elucidate research dynamics and collaborative patterns.

**Results:**

Annual MM drug resistance publications exhibited sustained growth, with a notable surge during 2022 to 2024. The United States (36.4%) and China (28.6%) dominated research output, while Harvard Medical School emerged as the most influential institution. Keyword clustering identified 5 core research domains: pharmacological interventions and therapies, resistance mechanisms and molecular pathways, cellular drug resistance mechanisms, tumor microenvironment and immunoregulation, and fundamental biology and diagnostics. Current research focuses on microenvironment-mediated resistance mechanisms and novel immunotherapies.

**Conclusion:**

This study delineates the intellectual landscape of MM drug resistance research, emphasizing key contributors, evolving priorities, and emerging frontiers. The findings offer actionable intelligence for guiding future investigations and evidence-based policymaking, potentially accelerating the development of optimized therapeutic approaches for chemoresistant MM.

## 1. Introduction

Multiple myeloma (MM) is a malignant hematologic neoplasm originating from bone marrow hematopoietic tissue, characterized by clonal proliferation of abnormal plasma cells. Early-stage disease often manifests asymptomatically, while advanced stages present with hallmark clinical features including osteolytic lesions, anemia, renal insufficiency, and immune dysfunction.^[1,2]^ Accounting for 1% to 1.8% of all malignancies and 15% of hematologic cancers, MM represents the second most prevalent hematological malignancy.^[3]^ Although therapeutic advances have improved survival outcomes in recent decades, a substantial proportion of patients still experience suboptimal survival rates and high relapse frequencies,^[4]^ posing significant challenges to global healthcare systems and socioeconomic development.

Current first-line therapies primarily combine proteasome inhibitors (PIs), immunomodulatory drugs (IMDs), and autologous stem cell transplantation.^[5]^ For elderly patients, melphalan-prednisone regimens combined with thalidomide or bortezomib remain standard-of-care.^[6,7]^ However, despite initial therapeutic responses, most patients develop progressive drug resistance, leading to diminishing survival benefits and escalating relapse rates. This emerging pattern underscores drug resistance as a critical barrier to clinical outcomes, demanding urgent global attention.

The molecular mechanisms underlying MM drug resistance involve multifaceted biological pathways. Glucocorticoid resistance correlates with functional defects in glucocorticoid receptors and upregulated pro-survival cytokines.^[8]^ PI resistance arises through proteasome system upregulation, PSMB5 mutations, and β5 subunit overexpression.^[9]^ Bortezomib-specific resistance associates with 8p21 chromosomal alterations,^[10]^ oxidative stress protein overexpression, and CXCR4 dysregulation.^[11]^ Daratumumab resistance mechanisms include CD38 downregulation coupled with CD55/CD59 upregulation.^[12]^

Current resistance-reversal strategies face clinical limitations. Verapamil and quinine demonstrated potential as chemosensitizers in VAD regimens, yet severe adverse effects restrict their utility.^[13,14]^ Cyclosporine analogs like PSC833 show improved p-glycoprotein binding affinity over cyclosporine D,^[15]^ though none have gained MM approval. Emerging approaches targeting nuclear factor kappa-light-chain-enhancer of activated B cells (NF-κB) signaling or heparanase-mediated extracellular signal-regulated kinase activation remain experimental.^[16]^ The complexity of MM resistance mechanisms necessitates systematic analysis of current research landscapes and predictive identification of novel therapeutic targets.

Bibliometric analysis provides quantitative assessment of academic literature distribution, structural relationships, and research trends, facilitating identification of knowledge frameworks, collaborative networks, and emerging hotspots.^[17]^ Using CiteSpace (College of Computing & Informatics, Drexel University, Philadelphia) and VOSviewer (Centre for Science and Technology Studies [CWTS], Leiden University, Leiden, The Netherlands) visualization tools, we analyzed Web of Science Core Collection data from 2015 to 2024 to map MM drug resistance research.^[18,19]^ To our knowledge, this constitutes the first bibliometric study in this domain. Our findings aim to guide future investigations into molecular mechanisms and pharmacological interventions, ultimately informing optimized clinical management strategies for MM.

## 2. Materials and methods

**Figure 1.**
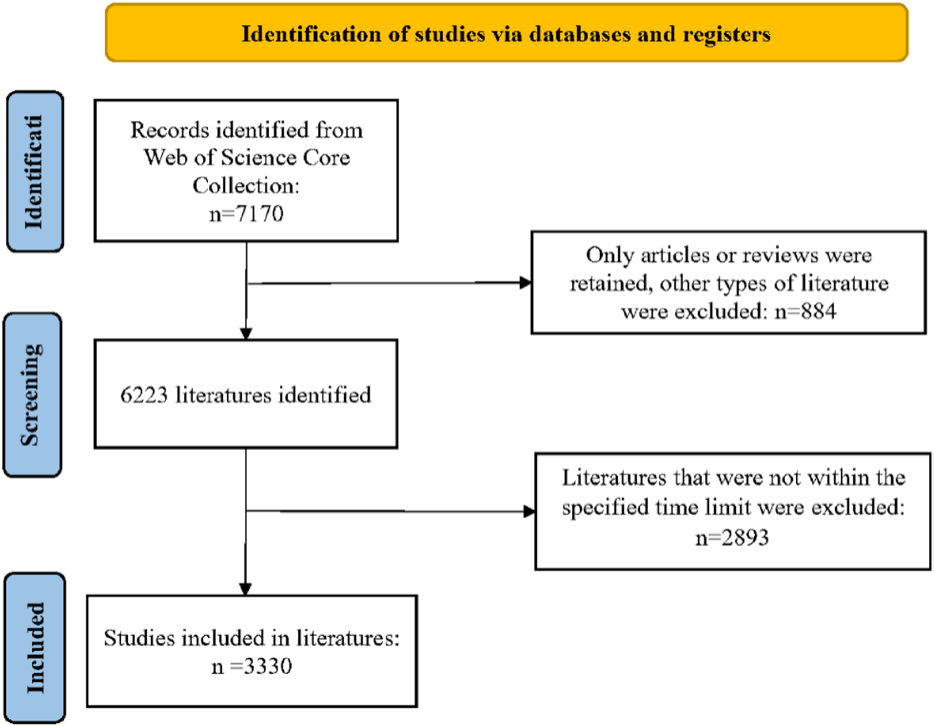
Retrieval flow chart.

Compared with other databases, Web of Science Core Collection demonstrates superior capabilities in visualizing analytical outcomes,^[20,21]^ which established its selection as the primary data source for this investigation. To minimize potential biases arising from database updates, all literature searches and data extraction procedures were completed on April 1, 2025. The search strategy was formulated as follows: ((((((TS = (myelomatosis OR “multiple myeloma” OR “plasma cell myeloma”)) AND TS = (resist* OR “drug resist*” OR “medicine resist*” OR “agent resist”)))) AND LA = (English)) AND DT = (Article OR Review)) AND PY = (2015-2024). The systematic workflow for literature retrieval and selection criteria is presented in Figure 1.

## 3. Results

### 3.1. Analysis of basic information

**Figure 2.**
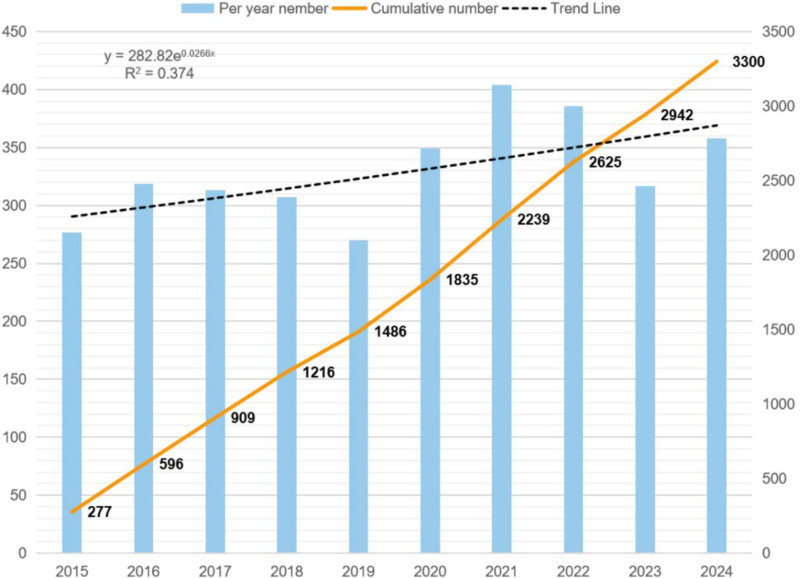
Literature publications during the decade of 2015 to 2024.

Figure 2 presents the annual publication trend analysis on MM drug resistance from 2015 to 2024, revealing a cumulative total of 3300 publications by 2024. Throughout this decade-long period, the annual publication output consistently exceeded 250 articles, with the most productive phase occurring between 2022 and 2024 when yearly publications surpassed 350 articles.

### 3.2. Analysis of countries/regions

**Figure 3.**
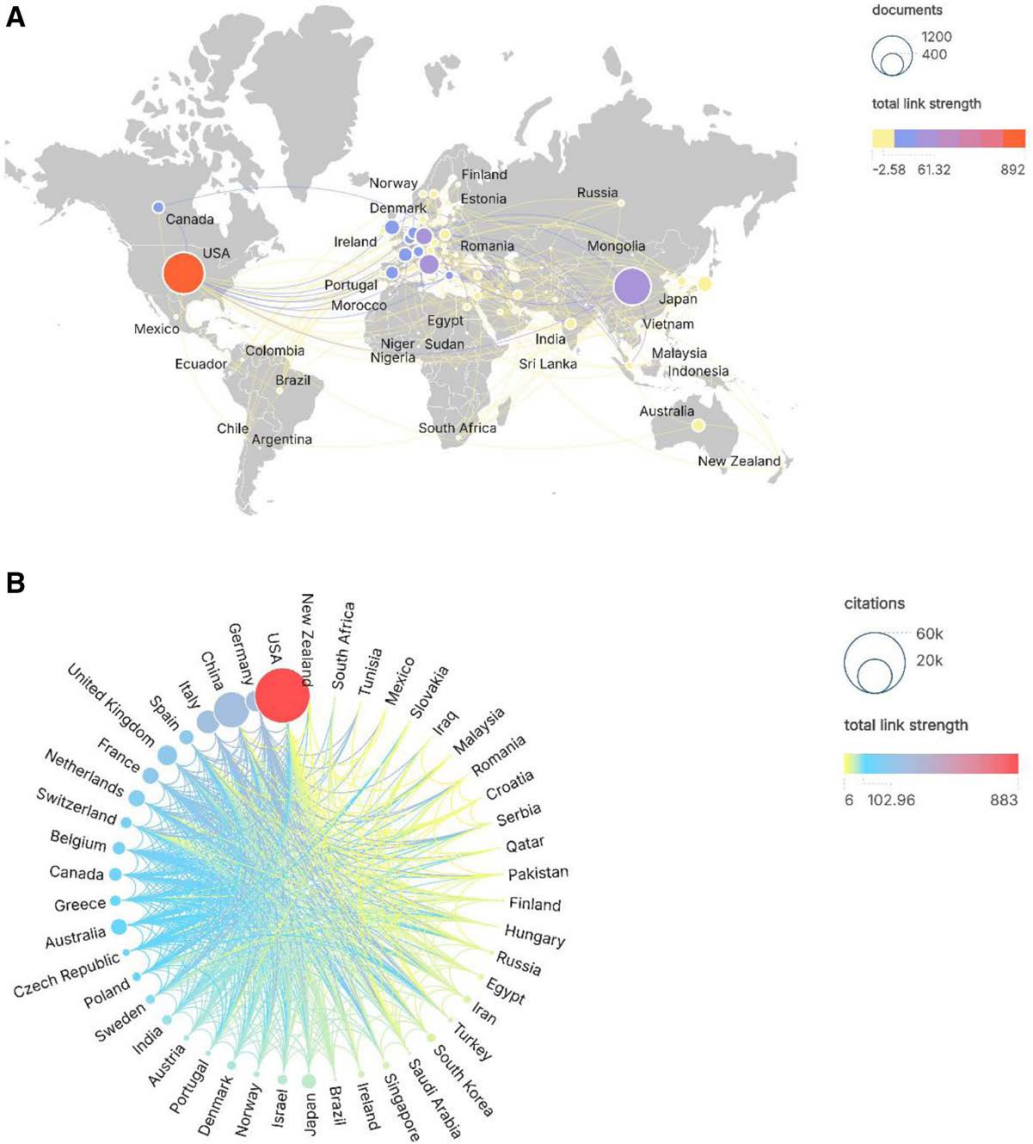
(A) World map visualization of global literature publications. (B) Global country citation and collaboration visualization.

As presented in Table 1, the United States leads in research output within this field with 1200 publications, accounting for 36.4% of total publications. China follows closely with 929 publications (28.6%), while other countries/regions all demonstrate publication outputs below 300. This study generated a global collaboration map concerning MM drug resistance using R software (R Foundation for Statistical Computing, Vienna, Austria) (Fig. 3A). Over the past decade, North America, East Asia, and Western Europe have made substantial contributions to this research domain. Additionally, Australia and Eastern Europe have also demonstrated notable research engagement.

**Table 1:** Ranking of top 10 countries involved in the relevant field.

Rank	Country	Publications	Centrality
1	USA	1200	0.26
2	Peoples R China	929	0.13
3	Italy	297	0.04
4	Germany	212	0.21
5	Japan	168	0.1
6	France	146	0.06
7	England	144	0.15
8	Spain	127	0.06
9	Australia	106	0.04
10	Netherlands	103	0.01

The participating countries/regions can be categorized into 4 tiers based on collaborative intensity: The first tier comprises North American countries/regions represented by the United States; the second tier consists of East Asian entities led by China; the third tier includes Western European nations exemplified by Germany, France, and the United Kingdom; while the final tier encompasses developing countries/regions such as India, African nations, and South American countries.

Furthermore, this investigation revealed intercountry/regional linkage strengths and total citation frequencies (Fig. 3B). The United States maintains dominant positions in both collaborative linkage strength and total citations, followed by Germany, China, and Italy. Notably, 2 countries exhibited total citations exceeding 20,000, while eighteen nations demonstrated collaborative linkage strengths surpassing 102.96 units.

### 3.3. Analysis of institutions/authors

As presented in Table 2, Harvard Medical School demonstrated exceptional research productivity with 155 publications and a centrality index of 0.41, securing its position as the global leader. Dana-Farber/Brigham and Women’s Cancer Center and Mayo Clinic were ranked second and third, respectively, also demonstrating remarkable academic influence. Other institutions exhibited fewer than 75 publications each, with relatively lower centrality indices requiring further improvement.

**Table 2:** Ranking of top 10 institutions involved in the relevant field.

Rank	Institution	Publications	Centrality
1	Harvard Med Sch	155	0.41
2	Dana Farber Canc Inst	90	0.16
3	Mayo Clin	75	0.18
4	Univ Texas MD Anderson Canc Ctr	72	0.11
5	Cent South Univ	52	0.02
6	Emory Univ	42	0.02
7	Soochow Univ	40	0.02
8	Univ Milan	40	0.11
9	Washington Univ	40	0.01
10	Univ Hosp Wurzburg	39	0.06

**Figure 4.**
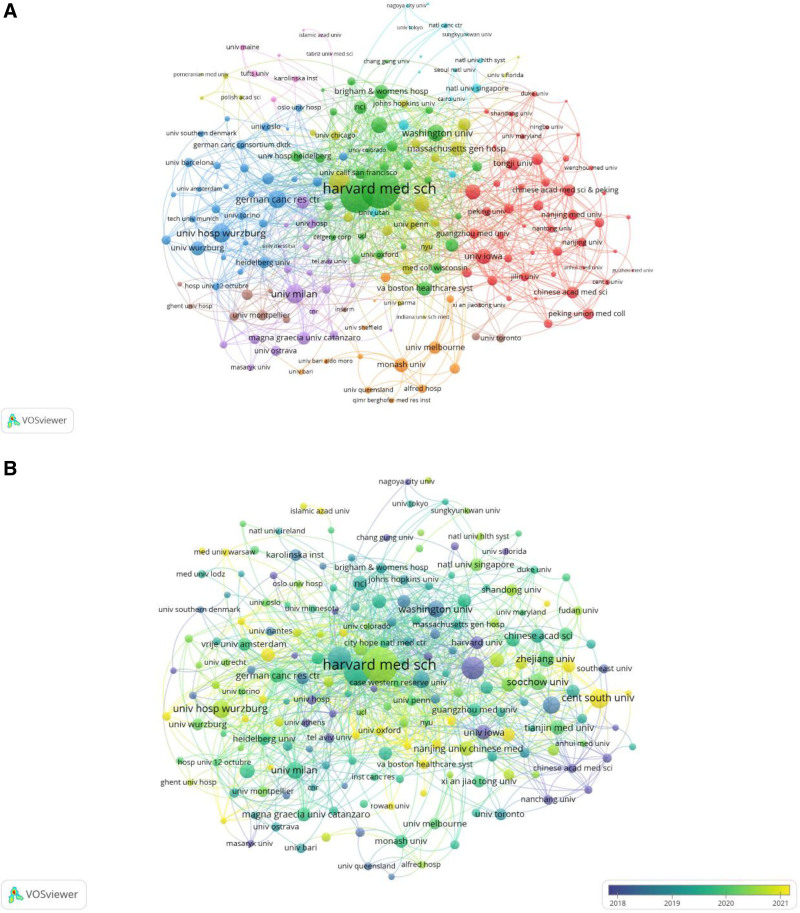
(A) Map of institutional cooperation networks with 10 or more publications. (B) Map of institutional cooperation networks with 10 or more publications (overlay time).

Figure 4A illustrates the classification of institutions with ≥10 publications into 5 major clusters. The blue cluster represents European institutions including the German Cancer Research Center and Würzburg University, while the red cluster comprises Chinese institutions such as Peking Union Medical College and Peking University. The green cluster features North American institutions led by Harvard Medical School. Furthermore, a time-based overlay network diagram was generated using VOSviewer (Fig. 4B), where purple and yellow hues denote early-stage and recent collaborations, respectively. Comparative analysis of Figure 4A and B reveals Harvard Medical School’s sustained academic dominance in MM drug resistance research, maintaining central positioning across temporal collaboration networks.

**Figure 5.**
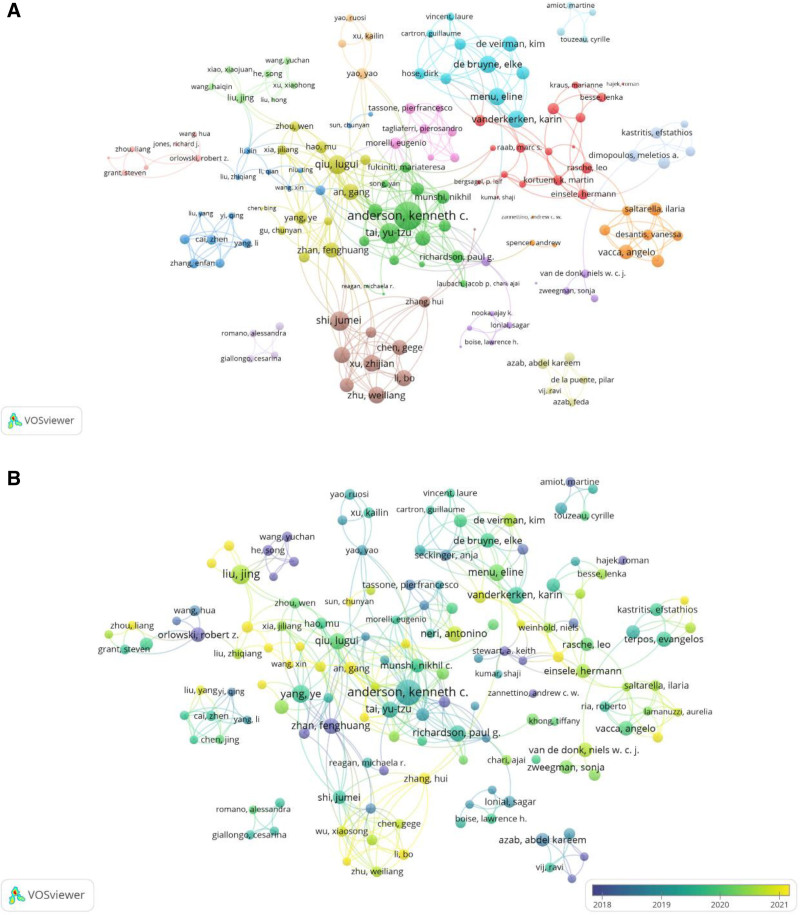
(A) Collaborative network diagram of authors with 10 or more publications. (B) Collaborative network map of authors with 10 or more publications (overlay time).

Table 3 summarizes author productivity and citation metrics, identifying Anderson Kenneth C. as the leading contributor with 64 publications. His substantially higher H-index and G-index values underscore his seminal contributions, establishing him as a pivotal researcher in MM drug resistance. The co-authorship network map (Fig. 5A) generated through VOSviewer analysis identified 15 distinct clusters. The primary green cluster was led by Anderson Kenneth C, followed by Qiu Lugui yellow cluster and Xu Zhijian brown cluster. The temporal author network (Fig. 5B) further confirmed Anderson Kenneth C persistent centrality, while revealing temporal activity patterns: scholars including Vij Ravi, He Song, and Zhan Fenghuang demonstrated early-stage prominence, whereas An Gang, Sun Chunyan, and Wang Xin emerged as more recent active contributors.

**Table 3:** Ranking of top 10 authors and cited authors with the most counts.

No.	Author	Count (%)	H-index	G-index	Cited author	Frequency
1	Anderson Kenneth C.	64	34	58	Anderson Kenneth C.	3492
2	Liu Jing	39	16	30	Van De Donk Niels W. C. J.	1855
3	Vanderkerken Karin	33	18	33	Tai Yu-Tzu	1689
4	Yang Ye	32	14	25	Orlowski Robert Z.	1467
5	Richardson Paul G.	30	19	30	Vanderkerken Karin	1273
6	Menu Eline	30	16	27	Zweegman Sonja	1202
7	Tai Yu-Tzu	28	21	28	Richardson Paul G.	1139
8	Einsele Hermann	28	17	28	Munshi Nikhil C.	1109
9	Qiu Lugui	28	15	28	Hideshima Teru	1028
10	Neri Antonino	27	15	24	Vacca Angelo	1002

### 3.4. Analysis of journals/areas

A statistical analysis of journals in this field was conducted to assess their academic productivity and impact (Table 4). The journals *Cancers* and *Oncotarget* demonstrated the highest publication outputs, with 175 and 108 articles, respectively. Meanwhile, *Blood* ranked first in citation frequency, accumulating 2873 citations.

**Table 4:** List of the top 10 journals and cited journals in the references.

No.	Journal	Frequency	Cited Journal	Citations
1	Cancers	175	Blood	2873
2	Oncotarget	108	Leukemia	2361
3	International Journal of Molecular Sciences	90	Cancer Res	2103
4	Frontiers in Oncology	88	Clin Cancer Res	2019
5	Leukemia	71	P Natl Acad Sci USA	1661
6	Blood	70	Oncotarget	1630
7	Blood Advances	55	Brit J Haematol	1621
8	British Journal of Haematology	55	Nature	1593
9	Frontiers in Immunology	47	New Engl J Med	1581
10	Clinical Cancer Research	45	J Clin Oncol	1534

**Figure 6.**
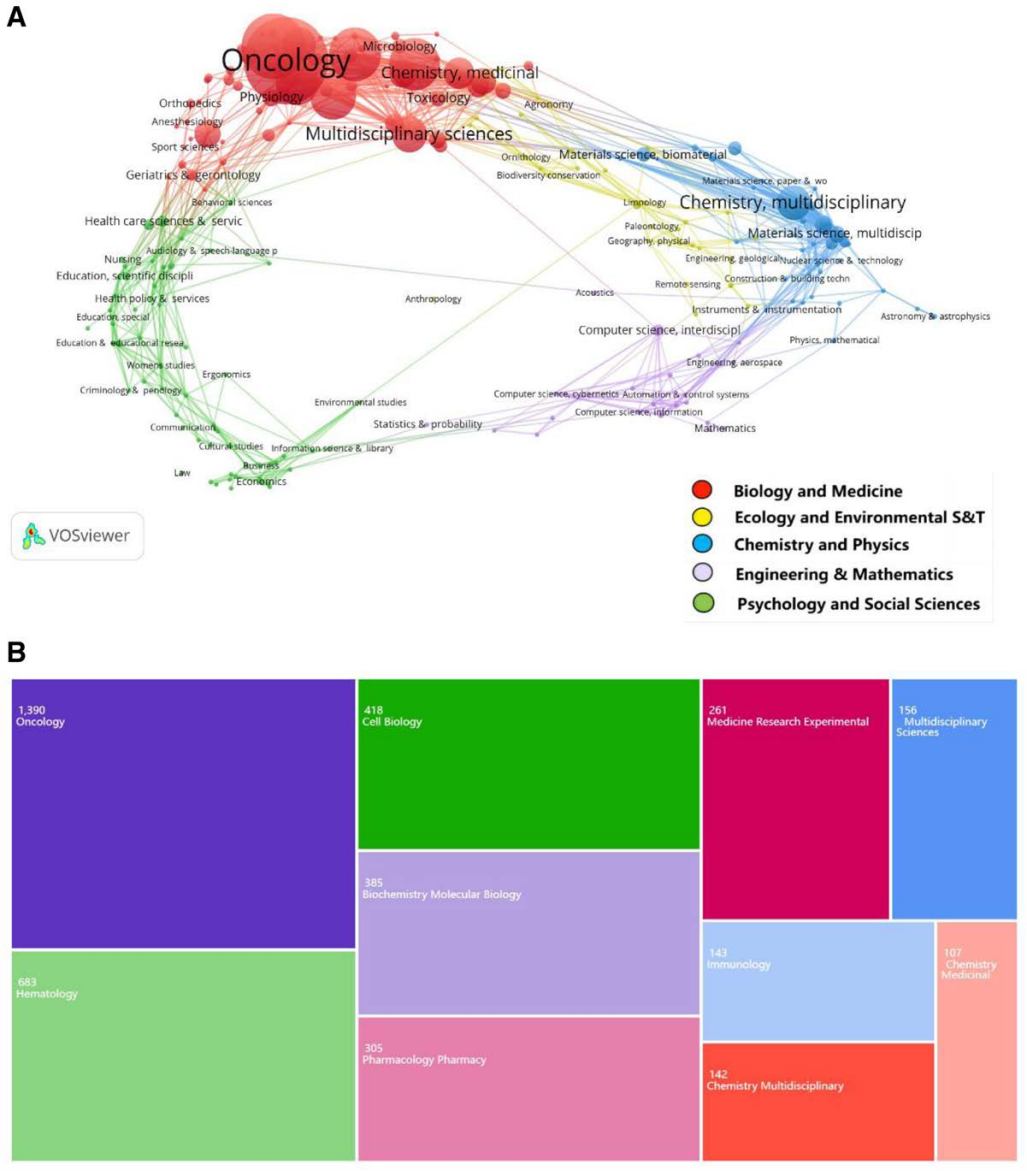
(A) Journal neighbourhood VOSviewer visual overlay map. (B) Visualization of the top 10 research neighborhoods.

As illustrated in Figure 6A, research domains in MM drug resistance were categorized into 5 distinct clusters: red (Biology and Medicine), yellow (Ecology and Environmental Science & Technology), blue (Chemistry and Physics), purple (Engineering & Mathematics), and green (Psychology and Social Science). The red cluster representing ``Biology and Medicine’’ emerged as the predominant research focus. Figure 6B displays the 10 most active disciplines in this field, with Oncology constituting the primary discipline, followed sequentially by Hematology and Cell Biology.

### 3.5. Analysis of keyword

Keywords serve as pivotal indicators of research hotspots and conceptual linkages within a scientific domain. This study presents the most frequently occurring keywords in the field, as detailed in Table 5. Centrality metrics quantify a keyword’s connectivity and hub function within co-occurrence networks, reflecting its bridging capacity across distinct research themes. Notably, “multiple myeloma” emerged as the keyword demonstrating both the highest occurrence frequency (*f* = 35) and network centrality (*C* = 0.78) in our analysis.

**Table 5:** The top 25 keywords of relevant research published between 2015 and 2024.

Rank	Keyword	Frequency	Centrality
1	Multiple myeloma	2040	0.12
2	Expression	632	0.04
3	Drug resistance	585	0.06
4	Resistance	476	0.05
5	Cancer	463	0.03
6	Bortezomib	362	0.05
7	Apoptosis	354	0.02
8	Cells	309	0.01
9	Therapy	277	0.03
10	Activation	255	0.04
11	Survival	237	0.02
12	In vitro	220	0.03
13	Dexamethasone	212	0.03
14	Bone marrow	208	0.06
15	Growth	201	0.03
16	Lenalidomide	196	0.03
17	Proteasome inhibitors	187	0.02
18	nf kappa b	177	0.02
19	Breast cancer	165	0.02
20	Open label	164	0.04
21	Proliferation	155	0.01
22	Inhibition	152	0.01
23	Antitumor activity	148	0.02
24	Pathway	135	0.01
25	Bone marrow microenvironment	134	0.02

**Figure 7.**
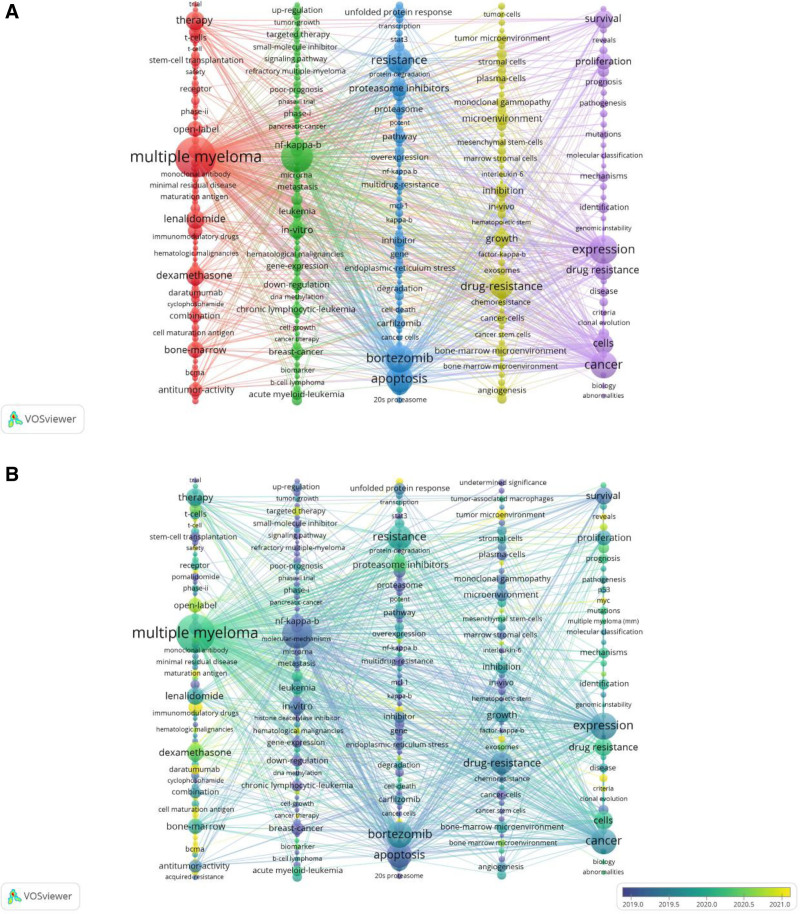
(A) Keyword clustering visualization. (B) Keyword clustering visualization (overlay time).

In this study, VOSviewer was employed to generate keyword cluster maps for this research domain (Fig. 7A). Distinct color schemes represent different thematic clusters, with node sizes corresponding to keyword frequency. The red cluster, designated as “Pharmaceuticals and Therapies,” contains high-frequency terms including “multiple myeloma,” “monoclonal antibody,” “lenalidomide,” “daratumumab,” and “dexamethasone,” focusing on clinical applications and safety assessments of existing therapeutic agents (e.g., IMDs, monoclonal antibody-based treatments).

The green cluster, categorized as “Drug Resistance Mechanisms and Molecular Pathways,” features prominent keywords such as “drug resistance,” “NF-κB pathway,” “signaling pathway,” and “gene expression,” highlighting investigations into molecular mechanisms underlying chemoresistance and genomic instability.

The blue cluster, identified as “Cellular Mechanisms of Drug Resistance,” demonstrates key terms including “apoptosis,” “resistance,” “proteasome inhibitors,” and “endoplasmic reticulum stress,” emphasizing cytological studies on resistance development.

The yellow cluster, labeled “Tumor Microenvironment and Immune Regulation,” aggregates terms like “tumor microenvironment,” “stromal cells,” “T-cells,” and “bone marrow microenvironment,” illustrating interactions between stromal/immune components and therapeutic resistance within neoplastic niches.

The purple cluster, characterized as “Fundamental Biology and Diagnostics,” incorporates terms such as “expression,” “mutations,” “prognosis,” “biopsy,” and “genomic instability,” underscoring molecular biomarkers and diagnostic approaches related to treatment resistance.

Figure 7B presents a time-overlay cluster map, revealing recently emerging keywords with high frequency including “targeted therapy,” “tumor microenvironment,” and “immunomodulatory drugs,” indicating current research trends.

**Figure 8.**
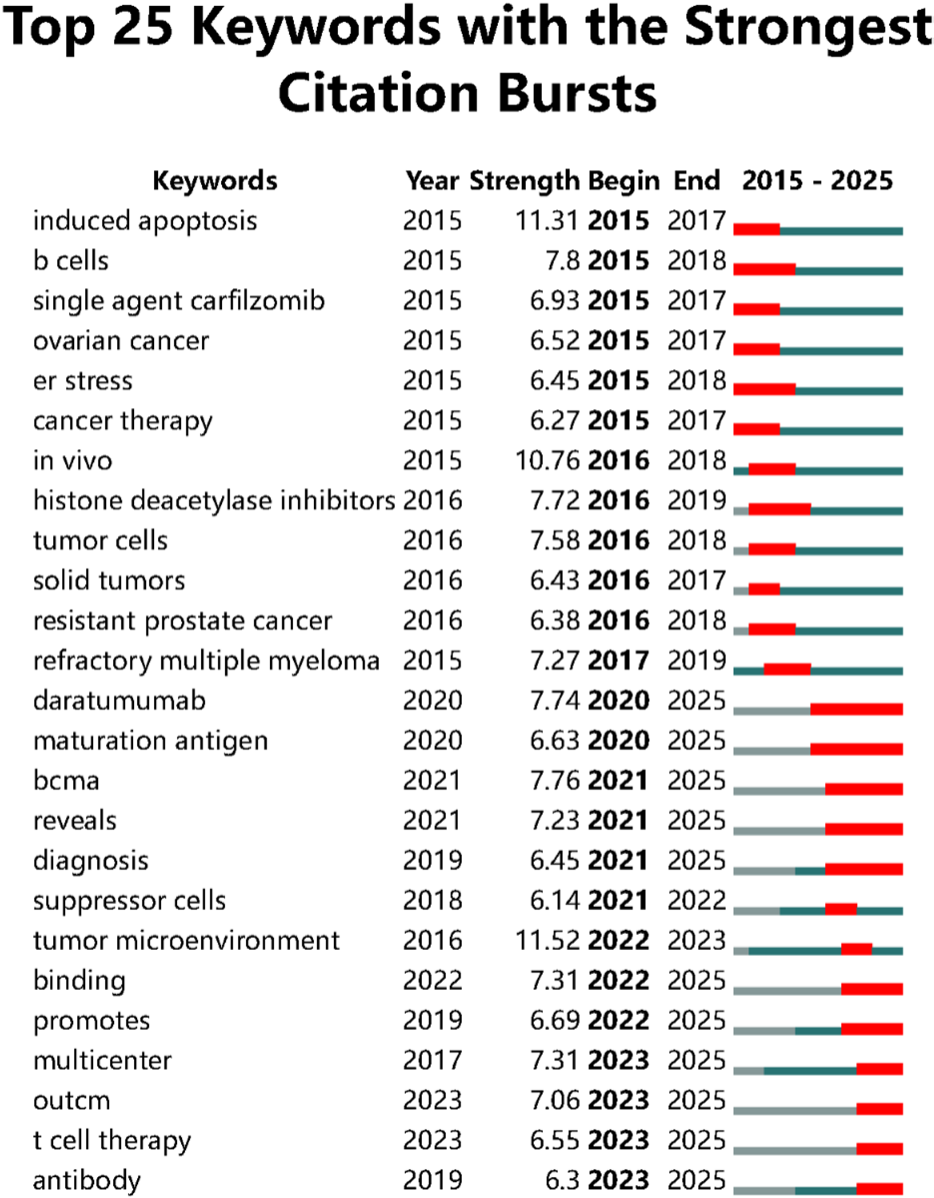
Top 25 keyword citation bursts.

The present study employed CiteSpace to generate a keyword burst graph, identifying the 25 most significant burst keywords in this research domain (Fig. 8). The initial burst keyword “induced apoptosis” emerged in 2015, while the majority of keywords exhibited burst activity predominantly after 2020. Notably, “tumor microenvironment” demonstrated the highest burst intensity among all identified terms. Several keywords including “Daratumumab” and “maturation antigen” remain in their active burst phase, indicating sustained research interest in these areas.

### 3.6. Analysis of literature

**Figure 9.**
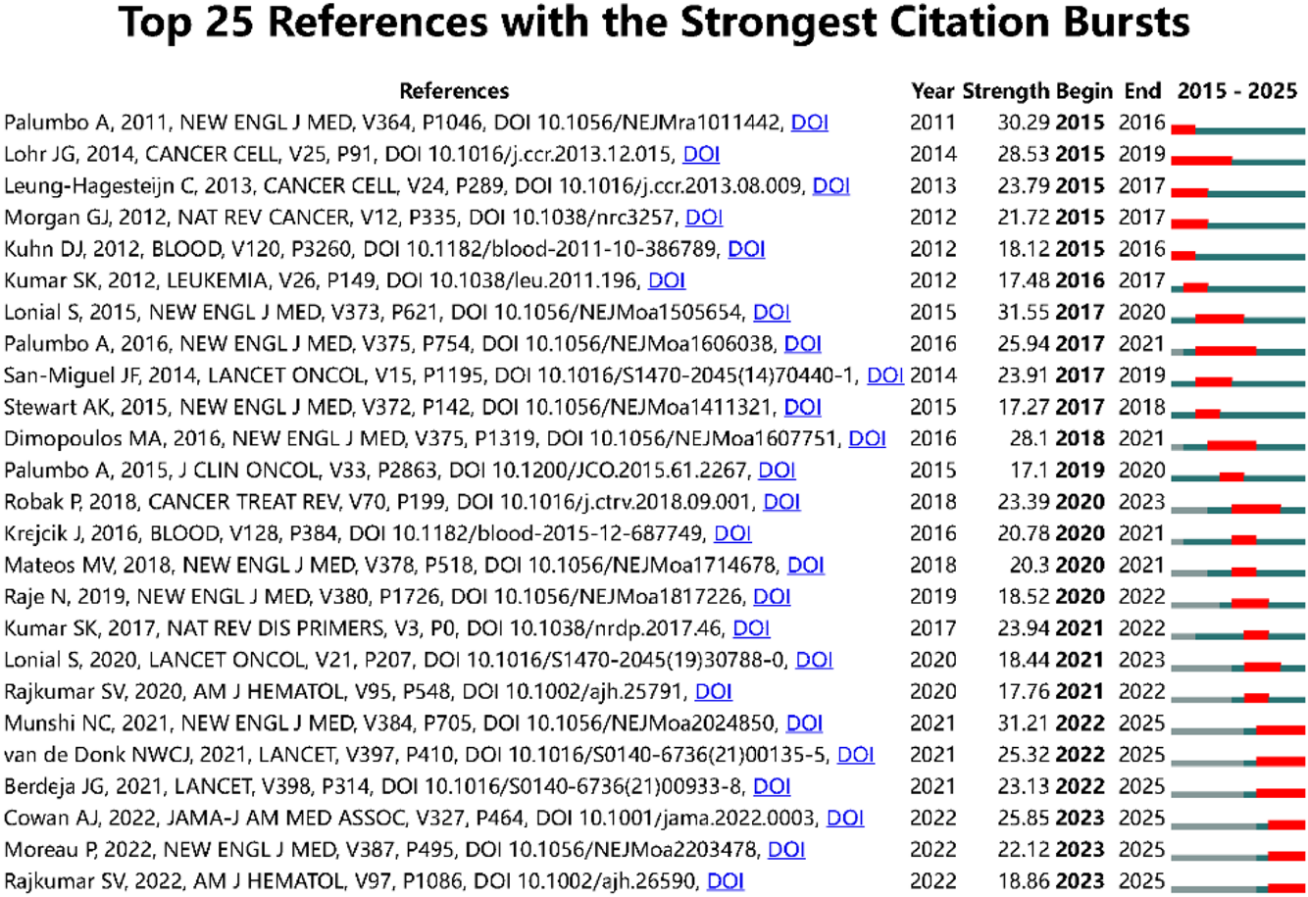
Top 25 citation bursts.

This study conducted a comprehensive analysis of the literature in this field to assist scholars in identifying seminal publications within the domain. As depicted in Figure 9, we employed CiteSpace to generate a citation burst graph, enabling the identification of the 25 most influential publications in the research area. The earliest seminal publication, authored by Antonio Palumbo et al, emerged in 2011.^[3]^ The majority of citation bursts occurred during and after 2017, with the study by Lonial et al demonstrating the most pronounced burst intensity in this research domain.^[22]^ In addition, there are currently 6 publications in the outbreak phase.

## 4. Discussion

### 4.1. Literature trends

As depicted in Figure 2, research on MM drug resistance continues to garner sustained global scholarly attention. The notable productivity from North America, East Asia, and Western Europe underscores their extensive engagement in this field. Concurrently, Australia, India, and the Middle East have also made substantial contributions to MM resistance research (Fig. 3A). To elucidate the collaborative nature of international investigations, we categorized participating countries/regions into 4 principal clusters. The red cluster, predominantly led by the United States, emerges as the most significant national/regional consortium. The United States maintains its leadership position through highly developed scientific resources and comprehensive research infrastructure, which have substantially propelled advancements in this domain.

Our analysis reveals a positive correlation between international collaboration intensity and citation frequency (Fig. 3B). The United States leads global rankings in both collaboration metrics and total citations, followed by China, India, and Germany. China demonstrates particular prominence as the second-ranked nation in both publication output and citation counts. Its robust collaborative networks with key research hubs in Western Europe and the United States position it to potentially emerge as the next focal point for scientific output, potentially forming a dual-core research framework with the United States in MM resistance studies.

This investigation highlights the critical importance of collaborative research environments in generating impactful scientific outcomes. The synergistic effect of international cooperation not only facilitates resource sharing and complementary strengths but also significantly accelerates the generation and dissemination of novel insights. Such collaborative dynamics provide substantial momentum for advancing mechanistic studies of MM drug resistance, offering crucial implications for understanding global research patterns and their impact on scientific progress.

Institutional and author contributions have played pivotal roles in advancing this field. The institutional analysis reveals that the green cluster led by Harvard Medical School emerges as the predominant institutional cluster (Fig. 4A). This finding underscores the importance of institutional collaboration in enhancing research productivity and impact. The integration of multidisciplinary perspectives has catalyzed innovative therapeutic strategies to overcome drug resistance, with collaborative approaches not only improving research efficiency but also significantly elevating the quality and translational potential of scientific outputs.

Chinese research institutions exhibit distinct collaborative patterns, with early-stage collaborations concentrated among institutions like Nanchang University and Southeast University, while recent collaborative networks predominantly involve Fudan University and Zhejiang University (Fig. 4B). These patterns may reflect regional disparities in medical development across China, suggesting that future leadership in MM drug resistance research may consolidate in regions with established medical infrastructure and funding advantages. Notably, recent intensified collaborations between European and American institutions likely correlate with aligned policy priorities, technological advancements, and evolving research trends.

The author clustering analysis identifies Kenneth C. Anderson green cluster as the central research consortium driving field development (Fig. 5A). Peripheral clusters featuring scholars like Ravi Vij and Ruosi Yao suggest potential diversification in MM research directions, indicating future opportunities for cross-team collaboration to enhance synergistic outputs. Pioneering contributors including Ravi Vij, Song He, and Fenghuang Zhan laid essential groundwork in early MM drug resistance studies (Fig. 5B). Current research momentum centers around Gang An, Chunyan Sun, and Xin Wang, whose investigations into emerging hotspots may yield critical breakthroughs in overcoming therapeutic resistance mechanisms.

Analysis of journal contributions and research domains provides additional insights into the structural landscape of this field. As summarized in Table 4, *Cancers* and *Oncotarget* emerge as the 2 most prolific journals in terms of publication volume, with publication counts of 175 and 108 articles, respectively. Notably, *Blood* demonstrates exceptional scholarly impact with 2873 accumulated citations, underscoring its scientific rigor and research quality within the domain.

Figure 6A delineates 5 principal research orientations in MM drug resistance investigation. The “Biology and Medicine” cluster predominates as the primary research focus, while significant contributions from “Chemistry and Physics” and “Psychology and Social Science” domains highlight the multidisciplinary nature of this field. This diversity underscores the necessity for extensive interdisciplinary collaboration in elucidating tumor resistance mechanisms and developing reversal strategies. The intensive cross-disciplinary interactions observed between “Biology and Medicine” and specialized subdisciplines including “Chemistry, Medicinal,” “Multidisciplinary Sciences,” and “Materials Science” potentially reflect current research hotspots. These may encompass investigations into drug resistance mechanisms, material science applications in MM drug delivery systems, and multidisciplinary therapeutic approaches. Future investigations exploring novel intersections between “Biology and Medicine” and “Engineering & Mathematics” domains may yield innovative contributions, particularly in developing computational models for drug resistance prediction.

Figure 6B reveals the core disciplinary composition of MM drug resistance research, with “Oncology” maintaining centrality. Moving forward, researchers should prioritize collaborative multidisciplinary approaches to decipher MM resistance mechanisms and develop reversal methodologies. Such integrative strategies are anticipated to foster scientific innovation and accelerate the translation of research findings into clinical applications.

The literature analysis has further elucidated pivotal contributions and emerging trends in this field. Published in Cancer Treatment Reviews, the review article by Pawel Robak et al systematically synthesizes clinical applications and mechanistic insights of targeted therapeutic agents, encompassing PIs, IMDs, and monoclonal antibodies, which continues to garner sustained scholarly attention within the research community.^[23]^ In addition, a randomized controlled trial conducted by Lonial et al demonstrated significant antineoplastic efficacy of the triple combination therapy comprising elotuzumab, lenalidomide, and dexamethasone, suggesting a potential paradigm shift in MM treatment strategies.^[22]^ Six articles currently experiencing citation bursts indicate emerging research frontiers in this field. The 2021 investigation by Nikhil et al revealed favorable response rates of idecabtagene vicleucel in patients with relapsed/refractory multiple myeloma.^[24]^ Concurrently, Berdeja et al reported that ciltacabtagene autoleucel achieved exceptional remission rates with manageable safety profiles in relapsed/refractory multiple myeloma populations.^[25]^ Subsequent research by Moreau et al in 2022 provided clinical evidence for teclistamab’s capacity to induce profound and durable responses in heavily pretreated myeloma patients.^[26]^ Rajkumar comprehensively summarized recent updates in annual diagnosis, risk stratification, and clinical management of MM in 2022,^[27]^ while Cowan et al systematically reviewed current diagnostic approaches and therapeutic strategies for this malignancy.^[28]^

### 4.2. Research hotspots and frontiers

The current study categorized key emerging terms from the past decade into 5 distinct clusters (Fig. 7A).

The red cluster (Pharmacological Interventions) highlights critical research on clinical applications and safety evaluations of established therapeutic agents (including lenalidomide, daratumumab, dexamethasone, and monoclonal antibodies) in MM resistance studies, emphasizing their therapeutic significance. The lenalidomide–daratumumab–dexamethasone triple regimen remains the first-line therapy for MM, demonstrating efficacy in prolonging overall survival and improving patient prognosis.^[29–33]^ However, emerging drug resistance patterns require urgent medical attention. Lenalidomide, a cornerstone immunomodulatory agent in MM management, has been implicated in resistance mechanisms. Through integrated proteomic, phosphoproteomic, and transcriptomic analyses, Yuen Lam Dora Ng et al identified CDK6 upregulation as a druggable target in lenalidomide-resistant MM.^[34]^ Daratumumab, a CD38-targeted monoclonal antibody, primarily exerts antitumor effects through antibody-dependent cellular cytotoxicity and complement-dependent cytotoxicity. Its resistance mechanisms involve CD38 downregulation coupled with upregulation of complement inhibitors CD55 and CD59.^[12]^ Future investigations should prioritize developing targeted therapies against CDK6, CD38, CD55, and CD59, potentially unveiling novel strategies to overcome tumor drug resistance. Green Cluster (Drug Resistance Mechanisms and Molecular Pathways) highlights the exploration of molecular mechanisms underlying drug resistance and genomic instability as pivotal research directions in this field. Vo et al demonstrated through integrated clinical sequencing that alterations in the NF-κB and RAS/mitogen-activated protein kinase signaling pathways occur in 45% and 65% of MM patients, respectively, with long-tail variants notably identified in the RAS/MAPK pathway.^[35]^ Dysregulation of the NF-κB pathway enhances tumor cell antiapoptotic capacity, which is closely associated with multidrug resistance and the autonomous adaptation of the bone marrow microenvironment.^[36]^ This underscores the importance of investigating key nodes within these signaling pathways to identify novel strategies for reversing therapeutic resistance. Blue Cluster (Drug Resistance Mechanisms and Cytological Studies) reflects significant advancements in cellular-level research. The 26S proteasome plays a critical role in MM progression and tumor proliferation. Proteasome inhibitors exert therapeutic effects through multifaceted downstream mechanisms, including suppression of NF-κB signaling, accumulation of misfolded/unfolded proteins (inducing endoplasmic reticulum stress and unfolded protein responses), downregulation of growth factor receptors, inhibition of adhesion molecule expression, and suppression of angiogenesis.^[37]^ Du et al revealed that targeting PSMD4/Rpn10, a ubiquitin receptor subunit of the 19S proteasome responsible for substrate delivery to the 26S proteasome, effectively suppresses tumor growth and overcomes PI resistance.^[38]^ These findings suggest that functional modulation of proteasome subunits or other cellular components may yield novel therapeutics to combat tumor resistance. Yellow Cluster (Tumor Microenvironment and Immune Regulation) emphasizes the research value of interactions between stromal/immune cells and drug resistance within the tumor microenvironment. Most MM patients develop drug resistance during disease progression, a phenomenon intricately linked to dynamic remodeling of the bone marrow microenvironment and tumor microenvironment (TME).^[39]^ Multi-omics studies by Cheng et al demonstrated progressive declines in neutrophils and mast cells during MM progression, alongside hyperactivation and exhaustion of NK cells within the TME. High-risk patients exhibited elevated CD8+ T cell proportions but reduced naïve CD4+ T cells and γδ T cells.^[40]^ These insights advocate for early intervention strategies to enhance neutrophil/NK cell cytotoxicity, tumor antigen presentation, and CD8+ T cell polyfunctionality, thereby reprogramming the immunosuppressive TME to disrupt tumor proliferation and improve therapeutic outcomes. Finally, Purple Cluster (Basic Biology and Diagnostics), representing molecular biomarkers and diagnostic approaches for drug resistance, outlines a critical roadmap for future investigations in this domain.

The persistent emergence of keywords such as “multiple myeloma,” “drug resistance,” and “poor prognosis” (Fig. 7B) highlights that chemoresistance-induced adverse prognosis remains an unresolved clinical challenge requiring further mechanistic investigation. Recently emerged terms including “targeted therapy,” “tumor microenvironment,” and “immunomodulatory drugs” suggest growing research focus on microenvironment-mediated drug resistance mechanisms and therapeutic efficacy of novel immunomodulatory agents.

Notably, “tumor microenvironment” demonstrates the highest burst intensity (Fig. 8), indicating its emerging prominence in contemporary research. Future investigations may prioritize elucidating microenvironmental dynamics influencing malignant cell chemoresistance. The sustained prominence of “daratumumab” reflects increasing scientific concern regarding CD38 monoclonal antibody resistance, which has evolved into a significant clinical dilemma. Additionally, emerging terms “maturation antigen” and “T cell therapy” suggest CAR-T cell therapies targeting B-cell maturation antigen may represent promising therapeutic strategies for refractory multiple myeloma. B-cell maturation antigen-directed therapeutic development is anticipated to become a major research frontier, potentially offering novel solutions for treatment-resistant cases.

### 4.3. Therapeutic strategies to overcome drug resistance in multiple myeloma

The inhibition of P-glycoprotein activity has emerged as a pivotal strategy to circumvent multidrug resistance (MDR). Studies demonstrate that cells exhibiting an MDR phenotype exhibit significantly enhanced responsiveness to chemotherapeutic agents (45% response rate) following treatment with MDR-reversing agents and membrane-active drugs, including calmodulin antagonists, calcium channel blockers, local anesthetics, and cyclosporine.^[41]^ Given that NF-κB activation contributes to chemoresistance, pharmacological inhibition of NF-κB represents another critical therapeutic approach for overcoming chemotherapy resistance in MM. Arsenic, a potent NF-κB inhibitor,^[42]^ has been shown to sensitize tumor cells to chemotherapeutic agents and enhance therapeutic efficacy.^[43]^

Antiapoptotic proteins such as Bcl-2 and Bcl-xL are implicated in resistance to chemotherapeutics including cyclophosphamide, methotrexate, melphalan, and corticosteroids.^[44]^ Notably, the Bcl-2 inhibitor venetoclax, currently approved for chronic lymphocytic leukemia, has demonstrated promising clinical activity in MM.^[45,46]^ Histone deacetylase 6 (HDAC6), which mediates aggresome-dependent protein degradation, has emerged as a therapeutic target for overcoming PI resistance. Recent studies indicate that coadministration of HDAC6 inhibitors can disrupt the clearance of misfolded proteins via the aggresome pathway, thereby overcoming resistance to PIs.^[47,48]^ The selective small-molecule HDAC6 inhibitor WT161 synergistically enhances bortezomib-induced cytotoxicity and reverses PI resistance. Combined treatment with bortezomib and WT161 in MM.1S cells elicits greater accumulation of polyubiquitinated proteins and activation of stress-activated protein kinase JNK compared to either agent alone.^[48]^ Furthermore, this combination demonstrates potent anti-myeloma activity in human MM xenograft models. The miR-221-222 cluster promotes tumorigenesis by downregulating tumor suppressors such as the pro-apoptotic p53-upregulated apoptosis regulator.^[49]^ Emerging evidence implicates this miRNA family in dexamethasone resistance mechanisms.^[50]^ Specifically, miR-221-222 overexpression confers dexamethasone resistance in MM.1S cells, while anti-miR-221-222 oligonucleotides partially restore dexamethasone sensitivity in resistant MM.1R cells. These findings suggest that pharmacological inhibition of miR-221-222 binding to p53-upregulated apoptosis regulator mRNA represents a potential therapeutic strategy to overcome dexamethasone resistance in MM patients.

## 5. Limitations

Due to discrepancies in data formats across databases, this analysis was restricted to studies indexed in the Web of Science core collection, potentially excluding relevant research from other sources. Additionally, the inclusion criteria limited selection to English-language publications, which may introduce geographical bias through the exclusion of non-English studies. Furthermore, inherent time lags between study publication and database indexing, coupled with the necessary citation accumulation period, might compromise the temporal currency of our findings in reflecting cutting-edge research developments.

## 6. Conclusion

In this study, we employed bibliometric analysis to examine global research trends and advancements in MM drug resistance over the past decade, offering critical insights for developing strategies to overcome tumor drug resistance. Our findings revealed that the United States and China emerged as the most prolific contributors in this field, having established prominent collaborative research networks. Harvard Medical School was identified as the leading research institution driving scientific progress. The analysis underscores the critical importance of international and interinstitutional collaboration in fostering innovative therapeutic approaches for MM drug resistance. Among academic journals, *Cancers* exhibited the highest publication output in this domain, while *Blood* emerged as the most frequently cited journal. Keyword clustering analysis revealed current research hotspots centered on 3 key areas: mechanisms of drug resistance, pharmacological interventions, and tumor microenvironment interactions. Collectively, this systematic investigation provides novel perspectives for addressing the multifaceted challenges associated with MM drug resistance, while mapping potential directions for future translational research.

## Author contributions

**Conceptualization:** Ye Gao, Zhengyang Li, Yuchen Mei.

**Data curation:** Xuanyu Yang, Fangzhen Lin, Siteng Zheng.

**Methodology:** Yifan Xie, Jiayu Ke, Ling Ling.

**Visualization:** Xuanyu Yang, Ye Gao, Zhengyang Li, Yuchen Mei.

**Writing – original draft:** Xuanyu Yang, Fangzhen Lin, Siteng Zheng.

**Writing – review & editing:** Xuanyu Yang, Fangzhen Lin, Siteng Zheng, Yifan Xie, Jiayu Ke, Ling Ling.
